# Synovial Tissue Biopsy Collection by Rheumatologists: Ready for Clinical Implementation?

**DOI:** 10.3389/fmed.2019.00138

**Published:** 2019-06-20

**Authors:** Marijn Smits, Sebastiaan van de Groes, Rogier M. Thurlings

**Affiliations:** ^1^Department of Rheumatology, Radboud University Medical Center, Nijmegen, Netherlands; ^2^Department of Orthopedic Surgery, Radboud University Medical Center, Nijmegen, Netherlands

**Keywords:** arthritis, synovial tissue, biopsy, clinical practice, implementation

## Abstract

Synovial tissue from arthritis patients is increasingly used for both basic pathophysiological and clinical translational research. This development has been spurred by the development of biotechnological techniques for analysis of complex tissues and the validation of ultrasound guided biopsies for easier tissue sampling. This increasing use of synovial tissue raises questions on standardization of methodologies for tissue processing and cellular & molecular analyses. Furthermore, it raises the question if synovial tissue biopsy analysis may be more widely implemented in clinical practice, what are the methodological hurdles for implementation and what are the lessons that can be learned from previous experience. This will be the focus of this review.

## Acquirement of Synovial Biopsies

There are several possible approaches to the acquisition of synovial tissue (1, 2). In most clinical practices tissue acquisition is performed by orthopedic surgeons at the operating theater, with the patient under sufficient anesthesia. For large joints arthroscopic biopsy is generally accepted as the gold standard, which gives a good quality and size of biopsy specimens in most cases (3). To acquire sufficient tissue from small joints an arthrotomy could be performed. During the past 25 years arthoscopic biopsy procedures have been increasingly used by academic rheumatological expert groups for basic pathophysiologic and clinical translational research. A number of their studies have addressed the minimal requirements for arthroscopic or ultrasound guided synovial tissue biopsies for scientific research.

In these studies the minimum number of biopsies to be retrieved was addressed. A minimum of 6 biopsies per procedure was shown to be sufficient to reduce sample variability in T cell numbers as analyzed by immunohistochemistry (2, 4–6). Other papers addressed the locations in the joint from which synovial biopsies should be acquired. It was found that macrophages and associated cytokines were unevenly distributed within the joint, while T cells and expanded T cell clones were more evenly distributed (7–9). The amount of synovial tissue needed depends on the clinical or translational questions and further research is needed for validation.

## Ultrasound-Guided Synovial Biopsies

A relatively new method to obtain synovial tissue is ultrasound (US) guided synovial biopsy, which is performed by trained rheumatologists. It can be performed by portal and forceps or Quick core needle. US biopsies are less invasive than arthroscopic biopsies and can be performed in both small and large joints (3, 6, 10–12). An advantage of US biopsy is that it is relatively easy to learn and it has a relatively small chance on side effects. A caveat is that synovial tissue yield is operator and index joint dependent and the operator needs to perform biopsies at regular intervals to retain skills to maintain a high success rate in obtaining good quality synovial tissue samples (6). The minimal requirements to retain skills is the subject of ongoing investigations. Furthermore, synovial tissue yield depends on the level of synovial inflammation as visualized by ultrasound. This seems to limits the application into research for conditions with low level of gray scale synovitis. Good quality synovial tissue was obtained from the knee in a cohort of RA patients in disease remission, but the success rate and tissue quality was not precisely reported (13). It has been shown that US guided synovial biopsies of joints selected on ultrasound parameters yield synovial tissue in 80–90% of cases of sufficient quality for histological evaluation and RNA extraction in both small and large joints (6). One study showed that the histological analysis of 2.5 mm^2^ from 4 biopsies of synovial tissue acquired by US biopsies is representative of the joint status in small joints of RA patients (14). In a recent multicenter retrospective study comparing arthroscopic biopsies with ultra-sound guided and blind needle biopsies on 159 procedures from 5 different academic rheumatology centers, there was no significant difference in the proportion of graded synovial tissue or total graded synovial tissue area and containing enough RNA of significant quality and quantity for transcriptomic analysis (15). These studies on tissue quality have only been investigated for a number of general assays, such as immunohistochemical staining of T cells and general retrieval of RNA. These diagnostic tests are not used in the clinical setting. Studies for these diagnostic tests have not been performed. It is therefore not precisely known what the density is of pathophysiological aberrations measured with various techniques or if there is an uneven distribution of biomarkers for different clinical conditions. Furthermore, if a number of diagnostic tests are combined within one patient, it is not known if the synovial tissue yield is similar between the first vs. later biopsies.

### Clinical Value of Synovial Tissue Sampling

Most clinical translational research focuses on prognostication and prediction of treatment response in patients with rheumatoid arthritis or psoriatic arthritis. To better understand the hurdles toward clinical implementation of potential biomarkers it is informative to critically appraise the use of synovial tissue diagnostic tests in current clinical practice. At this moment, synovial tissue analysis is infrequently used for differential diagnosis in patients with arthritis. There are many different causes of arthritis. For the rheumatologist it is frequently problematic to discriminate between these different causes. In a patient with arthritis the rheumatologist first analyzes the development in time and the number and pattern of involved joints. A major distinguishing factor for differential diagnosis is the presence of a mono- vs. oligo- or polyarthritis. Second, investigations such as imaging studies and blood tests may give additional clues for the cause. Also, examination of synovial fluid, when it is possible to aspirate this, can be of aid. Despite this, the rheumatologist can often not make a certain diagnosis (16). In most clinical practices synovial biopsies are performed by surgeons. Unfortunately, this can result in considerable delay. Sometimes, a biopsy is even omitted and patients are first treated with a trial of immunosuppressants and a biopsy is only performed if they do not respond. This can result in a prolonged period before an effective treatment is found with a long period of illness, invalidity and risk on permanent joint damage. Implementation of synovial biopsy sampling in these patients is also hampered by the relative limited amount of scientific reporting on this issue. In various case reports and series synovial biopsy analysis has shown an added value in addition to other diagnostic tests (17). From these reports it is however not entirely clear in which circumstances a synovial biopsy may precisely aid in diagnosis and what are the chances on sampling error. This is relevant because ultrasound-guided synovial biopsies are smaller compared to arthroscopic or arthrotomic tissue specimens. A careful reading of reported literature may give clues to the opportunities and hurdles for implementation in clinical practice for synovial biopsy analysis with existing diagnostic tests and this may also give insight into the hurdles for implementation of potential future diagnostic tests.

### Infectious Arthritis

There are many different pathogens that can infect synovial tissue. Below we discuss different causes of infectious arthritis and the value of synovial tissue analysis.

#### Acute Infectious Arthritis

Synovial tissue analyses can assist in the detection of joint infections (18). Most infections present as an acute onset mono-arthritis accompanied by fever. Less frequently, infectious arthritis presents as an indolent mono- or oligoarthritis. Causative organisms range from common gram-positive and gram-negative bacteria to *gonococci, Borrelia Burgdorpheri, mycobacteria, fungi*, or *Tropheryma Whippleii* infection. Synovial fluid culture yields growth of pathogenic bacteria in only a proportion of cases depending on the causative organism. Synovial fluid with a nucleated cell count ≥2,000 white blood cells/mm^3^ is considered inflammatory, the higher the leukocyte count (>10,000/mm3) and the greater the percentage of polymorphonuclear neutrophils (PMNs) (>90%), the higher the likelyhood of septic arthritis (19). Bacterial joint infections often have more than 75% of PMNs (20). In a recent study Coiffier et al. performed ultrasound guided synovial biopsies in patients with an acute monoarthritis (defined as <6 weeks duration). A total of 51 synovial biopsies were obtained from these patients from which 11 were positive on culture and defined as septic arthritis. Three of these biopsies had a positive synovial tissue culture and no bacterial growth on synovial fluid. This suggests it is useful to obtain synovial tissue in patients with an acute mono-arthritis and negative synovial fluid culture. Also the presence of perivascular infiltration of neutrophils in synovial tissue had a sensitivity and specificity of, respectively, 81.8 and 842% which leads to a likelyhood of 5,2 for the diagnosis septic arthritis (21).

*Neisseria gonorrhoeae* septic arthritis is often difficult to diagnose, for which mostly PCR or culturing on synovial fluid is performed. *N. Gonorrhoeae* is fragile and difficult to grow (22) The Gram stain reveals intra- and extracellular Gram-negative diplococci in <50% of cuture-positive fluids. Polymerase chain reaction (PCR) for *N. Gonorrhoeae* has a high specificity, which is estimated at 96–98% and a sensitivity of 78–80% (22). Broad-range bacterial primers to analyze genes coding for ribosomal RNA (16S rRNA) by polymerase chain reaction (PCR) may also show bacterial species (23–25). The available literature on the performance of these diagnostic tests mostly consists of case reports and series. It is therefore unknown in which cases and to what extent synovial tissue analysis is of added value compared to synovial fluid analysis. The use of 16S rRNA in diagnosis is for example under discussion since this test has also been reported positive in cases of rheumatoid arthritis and spondyloarthritis (25, 26). 16S rRNA analysis has also yielded positive results in uninfected liver and lymph node specimens. It is thought that this may be caused by amplification of RNA from bacterial fragments in endosomes of macrophages. In these cases 16S rRNA mostly yielded multiple organisms. Infectious arthritis might be characterized by the presence of rRNA from a single organism in multiple tissue specimens (27–29). A single study suggested that serial sampling could help in the decision to discontinue antibiotic treatment (29). However, the minimum amount of tissue that is required for immunohistochemical staining, culture and RNA analysis has not yet been systematically investigated.

#### Lyme Arthritis

A number of studies have focused on synovial tissue analysis in Lyme arthritis (23, 30–37). Lyme disease is a tick-borne infectious disease caused by different subspecies, most often *Borrelia Burgdorferi, B. Garinii, and B. Afzelii*. Lyme arthritis most common present as an intermittent or chronic mono-arthritis of the knee joint and less common an asymmetrical oligoarthritis (23). The causative agents and disease course and manifestations vary between continents. In the USA *Borrelia Burgdorferi* is the primary cause of Lyme disease (38). In Europe Lyme arthritis is most commonly caused by *B. afzelii, B. garini*, and *B. burgdorferi* occurs less often (39). About 60% of the untreated patients with Lyme disease develop Lyme arthritis as a manifestation of Lyme disease and about 10% of these do not respond to antibiotics (23, 36). Hypothetical explanations for this problem include the persistent presence of the organism or development of post-infectious inflammatory arthritis. *Borrelia Burgdorpheri* grows in blood and skin biopsies, but synovial fluid is a toxic environment for Borrelia species and successful cultivation is rarely seen (31, 32, 37). In spiked cultures adding small amounts of joint fluid results in rapid killing of spirochetes. For the diagnosis of Lyme disease it is recommended to use a two test approach for active disease and for previous infection using a sensitive enzyme immunoassay (EIA) or immunofluorescent assay (IFA) followed by a Western immunoblot. Negative EIA or IFA make a diagnosis of Lyme arthritis highly unlikely and remove the need for further testing (40). Lyme arthritis is a late stage of Lyme borreliosis and occurs several months after initial infection. Persons tested for Lyme disease almost always have a strong IgG positive response to *Borrelia Burgdorpheri* or blot antigens (41). However, positive serology may also reflect past (asymptomatic) Lyme infection.

PCR testing of synovial fluid for *Borrelia Burgdorpheri* DNA may be helpful for establishing a diagnosis of Lyme arthritis. There are different ways of PCR testing, qualitative PCR and quantitative PCR testing which detect different DNA sites encoding for *Borrelia Burgdorpheri* genes. Sensitivity of PCR testing on synovial fluid varies between 76 and 88% depending on which test is used in patients with clinical suspected Lyme arthritis and positive serology (30). Lyme arthritis can respond to antibiotic treatment despite a negative baseline Borrelia-PCR (23, 31, 32, 36). PCR-results vary, because technical execution is variable and different primer sets against different genes and subtypes of *Borrelia Burgdorpheri* are used. It is uncertain to what extent the sensitivity of Borrelia-PCR testing is diminished by cytotoxic effects of the synovial fluid on live Borrelia bacteria shed from the synovial tissue. Borrelia-PCR positivity often decreases after successful antibiotic treatment but may also persist. It persists more often in those with antibiotic refractory arthritis, but it may also disappear without further antibiotic treatment and does not correlate with time to remission in patients treated with DMARDs (33). This suggests that a persistent positive Borrelia-PCR test may result from either persisting living bacteria or prolonged but temporary presence of bacterial components in the absence of living bacteria in the synovial tissue.

Data on synovial tissue are limited. In two European studies Borrelia-PCR remained positive in the synovial tissue but negative in the synovial fluid in a small number of patients with Lyme arthritis persisting 2 months after antibiotic therapy. In one of these studies arthritis resolved post or propter additional antibiotic treatment (30, 34). In two USA studies Borrelia PCR was negative in all patients with antibiotic refractory arthritis 7–12 months after multiple antibiotic treatments (32, 33). In another study it was shown that susceptibility to antibiotic treatment differs between Borrelia subtypes so data between Europe and the USA may not be well comparable (31). Furthermore, it is uncertain if a positive Borrelia-PCR that persists in the synovial tissue despite antibiotic treatment reflects persisting live or dead/moribund bacteria. Other tests that may better reflect Borrelia viability, such as detection of Borrelia-mRNA, have been developed but not tested in this context (32). At the same time there is a lack of data on Borrelia species in the synovial tissue vs. fluid of patients with a persistent arthritis despite first-line antibiotic treatment. Overall, it can be clinically difficult to diagnose Lyme arthritis and to determine if the persisting arthritis is caused by persistent infection, post-infectious reactive arthritis or another rheumatological disease and challenging to manage the optimal duration of antibiotic vs. immunosuppressive treatment. Performance of current or new diagnostic tests in synovial tissue biopsies might be of added value, but this is uncertain.

#### Mycobacterial Arthritis

Tuberculous and non-tuberculous mycobacteria are an infrequent cause of arthritis and diagnosis is typically delayed from 5 to 50 months because of low initial clinical suspicion because of the very indolent onset, accounting 7% of all extrapulmonary tuberculosis (42) These patients most often present with a slowly progressive and destructive monoarthritis, mostly affecting knee and hip, while systemic symptoms can be absent. Chest radiography shows pulmonary involvement in around 50% of patients with osteoarticular tuberculosis. Tuberculin skin and quantiferon assay maybe falsely negative as a result from immunosuppression or natural waning of protective immunity. Ziehl-Nielsen is only positive in 10–20% of cases and cultures of synovial fluid in 80% and synovial tissue in 94% (42, 43). Histology showed caseating granulomatous inflammation in 90% of specimens, which can be hard to discriminate from granulomatous inflammation in other conditions including fungal joint disease, sarcoidosis, erythema nodosum, Brucellosis, Crohn's disease, and foreign body giant cell reaction (42). Diagnosis is made with PCR and/or culture in synovial fluid or tissue (24, 44). Synovial biopsy culture may be positive while culture of synovial fluid and blood is negative. In one series in 20% of all cases synovial biopsies were needed to detect *M. tuberculosis* (43). Mycobacterial infection may also result in a type of reactive oligo- or polyarthritis called Poncet's disease. In these cases it may be particularly challenging to discriminate infectious from reactive arthritis. Data lacks on the minimum amount of tissue to be acquired for the performance of relevant diagnostic tests.

*Mycobacterium leprae* can occur without cutaneous manifestations and present with articular features, mostly combined with neurologic involvement. Acute and chronic symmetric polyarthritis of hands, wrists, elbows and knees, and tenosynovitis are described. It may result from direct infiltration of the synovial membrane with *M. Lepra* bacilli or because of reactive arthritis. Occasionally, Lepra bacilli have been reported in synovial biopsies, but it has not been investigated how much synovium should be acquired to differentiate infectious from reactive arthritis (45).

Non-tuberculous mycobacteria (NTM) are very slowly growing bacteria and need special medium and prolonged incubation. PCR techniques are less sensitive but faster to diagnose NTM and can distinguish mycobacterium tuberculosis from non-tuberculous mycobacteria. Chronic granulomatous infection of tendon sheats, bursa, joints, and bone are most commonly caused by *Mycobacterium marinum, Mycobacterium avium intracellulare M kansasii, M terrae* complex, *M. Abscessus, M. Fortuitum*, and *M chelonae* most commonly seen in immune compromised patients. Surgical excision and antibiotic therapy is needed in these patients to prevent musculoskeletal damage (46, 47).

#### M. Whipple

Whipple's disease is caused by Tropheryma Whipplei, 65–90% presents with arthralgias. It typically presents as a chronic, often migratory and intermittent polyarthritis (48). It is most often accompanied by gastrointestinal complaints, signs of malabsorption, and in a proportion of patients, neurological, and cardiac complaints. A diagnosis is made by PAS staining and PCR from duodenal or jejunum biopsies, but has also been reported from blood, synovial fluid, or synovial tissue (48).

### Local Proliferative Conditions

Local proliferative and neoplastic conditions often result in abnormalities in conventional, ultrasound, or MRI images (49). However, these are absent in some cases, while specific pathological changes can be detected in the synovial tissue (50, 51). Synovial chondromatosis is a rare, benign condition that can occur as a primary condition but also secondary to joint damage. It involves metaplasia of synovial tissue into cartilaginous nodules. These gradually enlarge en eventually break loose to form intra- and periarticular loose bodies. These may ossify, continue to grow and induce tissue destruction. Especially at this later stage it may be hard to distinguish from intracapsular chondroma, chondrosarcoma, and there is a small risk on malignant transformation. Synovial tissue analysis may assist diagnosis both in very early stage and in late stage patients (52).

Pigmented villonodular synovitis (PVNS) is a benign disorder that involves hypertrophy of villonodular synovial tissue that gradually fills up the joint space. MRI typically shows a low signal on T1 and T2 eighted images because of hemosiderin content, but this may be masked by secondary synovitis, hemorrhage, or fat deposition. Based on imaging it may be difficult to differentiate from synovial sarcoma, recurrent hemarthrosis, or hemangioma. Synovial fluid may be bloody, xanthochromic, or clear. Synovial biopsy is considered the gold standard for diagnosis. It shows nodular fragments of hemosiderin and fat (53).

Synovial lipoma arborescens is a rare proliferative fatty process of the synovium. It may develop as a primary process or secondary to inflammatory or traumatic synovitis (54, 55). Synovial proliferation may also occur in response to a foreign body, such as surgery material, wood splinters, plant thorns, or sea urchin spine (56). Synovial biopsy may assist in diagnosis of these conditions in cases without clear etiology.

### Local Degenerative Conditions: Recurrent Hemarthrosis

Spontaneous recurrent hemarthrosis is a condition that can occur secondary to a number of conditions, such as osteoarthritis, torn lateral menisci, synovial proliferative lesions, or after arthroplasty. Cases caused by torn lateral menisci may be treated with meniscectomy and those with a synovial bleeding source by synovectomy or arterial embolization (57). Synovial tissue analysis shows hemosiderin depositions and may have assisted in diagnosis in isolated cases (58–60).

### Deposition Diseases

#### Gout, Pseudogout, Basic Calcium Phosphate Deposition Disease

Gout, pseudo-gout, and basic calcium phosphate deposition disease cannot always be diagnosed by synovial fluid analysis but can involve deposits of crystalline material in the synovial tissue (61–63). In case of suspected gout the tissue should be preserved with alcohol because the monosodium urate crystals can dissolve in other fixatives. Sections can be examined using a polarization microscope or using the DeGolanthal staining method. In a recent case series a group from Copenhagen University Hospital, Denmark, introduced the use of synovial biopsies to diagnose gout in patients without clinical arthritis or tophi. Biopsies were performed from MTP or ankle joints of 9 patients suspected of gout. Joints were selected that showed signs of gout on ultrasound, being intrasynovial hyperechogenicity, or articular double contours. Biopsy was performed with a sterile no-touch technique, as used for joint punctures, with an intra-muscular needle (21 gauge/0.8 mm). It showed synovial urate crystal deposition in 8 out of 9 patients (64). The authors argue that the 1 case in which no crystals were found might have been caused by sampling error. Synovial biopsies were also shown to assist in diagnosis of pseudogout patients with a seronegative polyarthritis (65). Basic calcium phosphate induced arthritis is hard to formally prove since the crystals are too small to be identified by (polarizing) light microscopy. They can be visualized using the calcium stain alizarin red S. A definite diagnosis can be made using transmission or scanning electron microscopy coupled with energy dispersive analysis, but this is mainly limited to the research setting.

#### Amyloidosis

Amyloid arthropathy results from deposition of immunoglobulin free light chains in patients with monoclonal gammopathies, multiple myeloma, or Waldenström's macroglobulinemia (66). It can manifest as joint and peri-articular soft tissue swellings or as arthritis. Most often it presents as a symmetric polyarthritis of small and large joints, but sometimes fewer or one joint may be involved (67). It may be the presenting symptom of multiple myeloma (68). Patients often have an increased erythrocyte sedimentation rate, Bence jones proteins in urine, anemia, hypercalcemia, and/or renal insufficiency. There can be clinical doubt whether the arthritis is caused by amyloid deposition in the presence of these clinical parameters. Amyloid deposits can be detected in the synovial tissue with Congo red staining with polarization microscopy and most sensitively fluorescent microscopy or immunohistochemical staining of light chains (69). Of 70 reported cases synovial biopsy was positive in 69 (99%) cases. In one case synovial biopsy was negative for amyloid and a subsequent renal biopsy was positive. In another an initial synovial biopsy was negative, but a subsequent synovial biopsy was positive. This indicates there sampling error may occur in this condition.

#### Hemochromatosis, Wilson's Disease, Ochronosis

Hemochromatosis involves arthralgia in a proportion of patients, which frequently involves a metocarpophalangeal osteoarthritis-like arthropathy. Sometimes a patient may present with episodes of acute arthritis of various joints that may be caused by pseudogout. Also case reports have been published of acute arthritis (70), apparently without signs of pseudogout, where synovial biopsies showed extensive cellular iron accumulation (71, 72).

Arthritis has been reported as a manifestation of Wilson's disease in isolated case reports. Synovial tissue X-ray energy spectroscopy of a synovial biopsy yielded the diagnosis in one case (73, 74).

Ochronosis is a rare genetically inherited metabolic condition that manifests as dark discoloration of the urine, dark pigmentation of the skin, and eyes and a progressive axial and peripheral degenerative arthropathy due to loss of cartilage integrity. The clinical manifestation and pathology results from joint replacement surgery sufficed for diagnosis in most reported cases, but synovial tissue biopsy might have assisted diagnosis in some cases. It shows necrotic, brown cartilage debris, and sometimes foreign body type reactions including histiocytes and giant cells containing ochronotic material (75).

### Systemic Proliferative Conditions

Rare systemic proliferative non-infectious conditions and neoplastic conditions such as histiocytositic conditions, sarcoidosis, melanoma, leukemia/lymphoma, and metastasis often can be diagnosed based on pathological changes in other tissues or organs, but these sometimes lack and typical synovial tissue pathological changes may yield a diagnosis (76–78). Histiocytic conditions, such as multicentric reticulohistiocytosis, Langerhans cell histiocytosis, and Erdheim–Chester disease, typically involve tissue infiltration of bones, the reticuloendothelial system and various organs (79–83). They have been associated with mono-, oligo-, and polyarthritis and synovial biopsy has assisted in differential diagnosis in multiple reported cases. It typically shows infiltration by disease associated histiocyte subtypes and various subset of giant cells (17, 84–86).

## Opportunities and Hurdles for Clinical Implementation of Synovial Tissue Analysis by Rheumatologists

### Opportunities

Taken together, the validation of ultrasound guided synovial biopsies and development of novel potential diagnostic tests offers an opportunity for synovial biopsy analysis by rheumatologists. This is relevant for patients with arthritis in whom synovial tissue analysis is considered, since tissue acquisition is currently generally performed by surgeons. This may lead to a considerable delay. There especially seems to be an indication for a synovial biopsy in patients with a monoarthritis where blood, synovial fluid, X-ray and MRI investigations yield insufficient clues. Still, the jury is out whether a rheumatology center can best invest in an efficient referral system to their surgical or radiological colleagues or start performing these biopsies themselves.

### Hurdles

There seem to be some hurdles for implementation of ultrasound guided biopsies. Case studies concern relatively rare etiologies and these vary between countries. Furthermore, the technical approach and analytic yields vary. Besides, the reports often lack full description of other diagnostic clues. Most importantly, there is a lack of systematic prospective investigations in at risk populations. Therefore, it is controversial how often a synovial biopsy is of added value. It is also not known if ultrasound guided biopsy can reliably substitute arthroscopic or arthrotomic procedures, especially when multiple tests need to be performed. In a recent case series of 74 patients with undifferentiated arthritis by Najm et al. (16) synovial biopsy analysis was performed with ultrasound guided biopsies of large and small joints in a number of rheumatological expert centers in France. 58 patients had an acute or chronic monoarthritis, 7 an oligoarthritis, and 6 a polyarthritis. Biopsy size was assessed sufficient if larger than 0.5 mm^2^ based on previous literature assessing heterogeneity of histology in RA (16). The biopsies were of sufficient quality in 82% of patients, the yield depended on learning curve and joint accessibility. These allowed a definite diagnosis in 16% of the patients. Five patients underwent a secondary arthroscopy/-tomy because of suspicion of a septic arthritis which yielded a diagnosis of pseudogout in one patient. A case of Lyme and Whipple were diagnosed based on PCR in 2 patients (16). These data are promising but a number of questions have not yet been systematically addressed:

What is the number of procedures that should be performed yearly to retain skills in routine clinical practice? What is the minimum of synovial biopsies that should be taken for each diagnostic test, especially in patients in whom multiple tests need to be performed (6)? Should different joint sites be biopsied to exclude specific conditions, such as Borrelia, which might have a predilection for initial infection of the hamstring tendons (45)? What is the best quality control to ensure that synovial instead of other joint tissue is acquired for culture or RNA analysis?

## Conclusion

Analysis of synovial biopsies has been extensively validated for experimental research and increasingly for clinical translational research and clinical practice. Further concerted international collaboration is needed to understand the utility of synovial biopsies in clinical decision making in patients with mono- oligo-, or polyarthritis in the context of other clinical clues. Furthermore, the technical constraints of ultrasound guided biopsies need to be studied in comparison with the gold standard: surgical biopsies. Participation in research networks or quality registries is essential for successful clinical implementation.

## Author Contributions

All authors listed have made a substantial, direct and intellectual contribution to the work, and approved it for publication.

### Conflict of Interest Statement

The authors declare that the research was conducted in the absence of any commercial or financial relationships that could be construed as a potential conflict of interest.
